# Colon cancer care and survival: income and insurance are more predictive in the USA, community primary care physician supply more so in Canada

**DOI:** 10.1186/s12939-015-0246-z

**Published:** 2015-10-29

**Authors:** Kevin M. Gorey, Sindu M. Kanjeekal, Frances C. Wright, Caroline Hamm, Isaac N. Luginaah, Emma Bartfay, Guangyong Zou, Eric J. Holowaty, Nancy L. Richter

**Affiliations:** School of Social Work, University of Windsor, 401 Sunset Avenue, Windsor, Ontario N9B 3P4 Canada; Department of Oncology, Windsor Regional Cancer Center and Schulich School of Medicine and Dentistry, Western University, London, Ontario Canada; Division of General Surgery, Sunnybrook Health Sciences Center and cross appointed Departments of Surgery and Health Policy, Management and Evaluation, University of Toronto, Toronto, Ontario Canada; Department of Geography, Western University, London, Ontario Canada; Faculty of Health Sciences, University of Ontario Institute of Technology, Oshawa, Ontario Canada; Department of Epidemiology and Biostatistics and Robarts Research Institute, Western University, London, Ontario Canada; Dalla Lana School of Public Health, University of Toronto, Toronto, Ontario Canada; School of Social Work, University of Windsor, Ontario, Canada

**Keywords:** Health insurance, Poverty, Colon cancer care, Survival, Primary care physicians, Gastroenterologists, United States, Canada, Health care reform, Patient protection and affordable care act

## Abstract

**Background:**

Our research group advanced a health insurance theory to explain Canada’s cancer care advantages over America. The late Barbara Starfield theorized that Canada’s greater primary care-orientation also plays a critically protective role. We tested the resultant Starfield-Gorey theory by examining the effects of poverty, health insurance and physician supplies, primary care and specialists, on colon cancer care in Ontario and California.

**Methods:**

We analyzed registry data for people with non-metastasized colon cancer from Ontario (*n* = 2,060) and California (*n* = 4,574) diagnosed between 1996 and 2000 and followed to 2010. We obtained census tract-based socioeconomic data from population censuses and data on county-level physician supplies from national repositories: primary care physicians, gastroenterologists and other specialists. High poverty neighborhoods were oversampled and the criterion was 10 year survival. Hypotheses were explored with standardized rate ratios (RR) and tested with logistic regression models.

**Results:**

Significant inverse associations of poverty (RR = 0.79) and inadequate health insurance (RR = 0.80) with survival were observed in the California, while they were non-significant or non-existent in Ontario. The direct associations of primary care physician (RRs of 1.32 versus 1.11) and gastroenterologist (RRs of 1.56 versus 1.15) supplies with survival were both stronger in Ontario than California. The supply of primary care physicians took precedence. Probably mediated through the initial course of treatment, it largely explained the Canadian advantage.

**Conclusions:**

Poverty and health insurance were more predictive in the USA, community physician supplies more so in Canada. Canada’s primary care protections were greatest among the most socioeconomically vulnerable. The protective effects of Canadian health care prior to enactment of the Affordable Care Act (ACA) clearly suggested the following. Notwithstanding the importance of insuring all, strengthening America’s system of primary care will probably be the best way to ensure that the ACA’s full benefits are realized. Finally, Canada’s strong primary care system ought to be maintained.

## Background

Our research group has studied cancer care among the poor in the USA and Canada and consistently observed Canadian advantages [1–5]. In fact, the more impoverished the people and places the larger the Canadian advantages. For example, we studied breast cancer in the poorest neighborhoods of California and Ontario [6–8]. Five-year survival rates differed by 20 percent or more, universal health insurance accounting for most of the Canadian advantage [1, 9]. Our premise has been that focusing on the experiences of the most vulnerable people in the most vulnerable places magnifies clinical, policy and human significance.

The late, preeminent primary care researcher and advocate, Barbara Starfield, commented that “insurance is a necessary, but not sufficient explanation” [10–12]. She theorized that Canada’s greater primary care-orientation is also significantly protective. Her theory is consistent with our observation of much greater primary care physician (PCP) representation among the physician workforce of Ontario (47 %) than California (27 %) [13]. A combined Starfield-Gorey theory of Canada-USA health care postulates the following. First, personal income and health insurance, well-known to be highly predictive of health care quality in the USA, matter less in Canada’s guaranteed-access system. Second, community health care endowments, especially the supply of PCPs, matter more in Canada.

We tested the Starfield-Gorey theory with a historical study of breast cancer care in California and Ontario during the pre-Affordable Care Act (ACA) era [Gorey KM et al., unpublished observations, 2015]. The first Canada-USA study to examine the independent effects of poverty, health insurance and PCP supply, it’s theoretically consistent highlights follow. The association of poverty with suboptimum care was strong in the USA, but nonexistent in Canada. The association PCP supply with optimum care was stronger in Canada than in the USA, and the Canadian advantages were completely explainable by better health insurance and primary care. Notwithstanding the importance of insuring all Americans, this study suggested a way to further ensure ACA protections, that is, increase the supply of PCPs. However, it was only one test of the theory. The present study is a systematic replication with another important health care quality indicator, colon cancer care.

Sensitive to social and economic forces, colon cancer care is a useful quality sentinel for comparing health care systems. The second leading cause of cancer death in North America, its prognosis can be excellent with early diagnosis and treatment [14–17]. Colon cancer screening and investigative technologies as well as surgical and systemic treatments that matter in terms of survival have proliferated in Canada [18–22] and the USA [23–30] with moderate to strong inverse socioeconomic associations in the USA, but modest to nil or null ones in Canada. Community PCP supply has been associated with earlier diagnosis as well as better treatment and survival in both countries [13, 31, 32]. However, because of disparate analytic models and lack of control for potentially potent confounds, the relative size of PCP supply-colon cancer care and survival effects in each country are not yet known. The few studies of specialist physician effects have been less consistent. Two studies in the USA suggested that increased specialist physician supplies may lead to disparities in colon cancer care as well as to other health disparities [32, 33]. Quite the opposite was observed in a Canadian study that found a protective effects of gastroenterologist supply [13]. We are not aware of any Canada-USA study of colon cancer care that has examined the independent effects of poverty, health insurance and physician supplies, PCPs and specialists. This controlled observational study will do so.

## Methods

### Study samples

We oversampled people who lived in poverty and were diagnosed with colon cancer in Ontario and California between 1996 and 2000 and followed them until enactment of the ACA in 2010. Cohorts were 4,574 people in California and 2,060 people in Ontario with the most treatable, non-metastasized colon cancers. Those diagnosed with stage IV disease according to the American Joint Committee on Cancer were excluded [34]. Ontario and California are among places with the most comprehensive and valid colon cancer registries in the world [35–37]. National death indexes were used by both registries.

### Poverty, health insurance and physician supply measures

Since neither Ontario nor California registries collect income data, we joined them to neighborhood data via Statistics Canada (2001) and USA (2000) census tracts [38, 39]. A third of participants in California were randomly selected from high poverty neighborhoods where 30 % or more of the people were poor. Others were randomly selected from middle (5-29 % poor) and low poverty neighborhoods (<5 % poor). The Ontario sample similarly overrepresented the lowest income neighborhoods and then randomly selected from middle and high income neighborhoods. Lowest income neighborhoods in the two countries were quite similar. Median annual household incomes in US dollars were only slightly lower in California ($23,125) than Ontario ($24,175) [40]. Primary health insurers were defined in California as private (included Medicare with private supplemental coverage), public (Medicare alone or Medicaid) or none.

We then identified communities with relatively low to high health care endowments characterized by physician supplies. To do this we joined participants to county-level active physician data via the American Medical Association and Canadian Institute for Health Information databases (2000–2001) [41–44]. PCPs were those who reported their specialty area as general or family practice. Consistent with validated definitions and practice patterns, general internists in the USA and emergency family medicine physicians in Canada were also included as PCPs. Physicians reporting the majority of their time in specialized practice or who were board certified in that specialty were so defined [45, 46]. Threshold effects, below which the participants were less likely to survive, were identified by exploring 0.5 physician increments [47]: < 7.5 PCPs per 10,000 or 2 gastroenterologists (GE) per 100,000 community inhabitants. Thresholds were not observed for the supplies of overall specialist physicians or for other specialists often involved in colon cancer care such as general surgeons or oncologists.

### Analysis

According to the Starfield-Gorey theory poverty is a strong predictor of early death in the USA, while adequate PCP supply better predicts long term survival in Canada. These hypotheses involve interaction effects: poverty-by-country and PCP supply-by-country. We used a logistic regression model to test them [47]. We estimated the strength of each predictor-10 year survival relationship (e.g., increased survival odds associated with PCP supply increases) adjusting for the effects of other predictors as well as potential confounds. We accounted for stage as well as initial surgery and chemotherapy which were available in California, but that we had to retrospectively collect from health records across Ontario at considerable cost. Therefore we oversampled California participants to serve as multiple “controls.” A strategy to efficiently increase analytic power, it meant that we could detect rate differences as small as 5 % (2-tailed α = 0.05; power_1 - β_ = 0.80) [48]. All variables, except three, had less than 1 % missing data: number of regional lymph nodes examined (2.4 %), wait time for treatment (5.4 %) and tumor grade (8.9 %). Odds ratios (OR) and confidence intervals (CI) were estimated from regression statistics.

We also described significant interactions by comparing within and between-country survival rates across poverty and physician supply strata. We directly adjusted rates using a standard population with the age and stage characteristics of this study’s combined Ontario-California sample. It was used to calculate age and stage-standardized rates for the Ontario and California samples. All rates were calculated per 100 participants and reported as percentages. We used rate ratios (RR) for comparisons with 95 % CIs derived from the Mantel-Haenszel *χ*^2^ test. Further methodological details have been published [13, 16, 49–52].

## Results

Physician supply statistics are displayed in Table 1. First, the supply of PCPs was greater in Ontario. There was 1 more PCP for every 10,000 Ontarians than Californians (means of 7.8 and 6.8). Moreover, 4 of every 10 participant in Ontario (39.6 %), but only 1 of every 10 in California (11.9 %) lived in adequately supplied communities with 7.5 or more PCPs for every 10,000 inhabitants. Second, the supply of specialists was much greater in California, where there were 10 more specialist physicians for every 10,000 inhabitants than in Ontario (means of 17.8 and 7.5). Third, the supply of GEs was much greater in California. There were 2.4 more GEs for every 100,000 Californians than Ontarians (means of 3.2 and 0.8). Additionally, nearly all in California (86.4 %) and few in Ontario (4.7 %) lived in adequately supplied communities with at least 2 GEs per 100,000 inhabitants. Finally, the pattern for other specialists was similar.

**Table 1:** Physician densities in communities where people with colon cancer resided in California and Ontario, 2000

Physician specialization community density	California		Ontario	
	Sample	Percentage	Sample	Percentage
Primary care physicians per 10,000 inhabitants				
1.9 to 5.9	2,003	34.7	1,000	37.0
6.0 to 7.4	3,086	53.4	631	23.4
7.5 to 18.0	687	11.9	1,069	39.6
	*M* = 6.8 (*SD* = 2.0)		*M* = 7.8 (*SD* = 2.2)	
	*Mdn* = 6.8 (range: 0.0–18.0)		*Mdn* = 7.3 (range: 5.00–15.0)	
Specialist physicians per 10,000 inhabitants				
0.0 to 9.9	839	14.0	2,112	78.6
10.0 to 19.4	2,189	37.0	576	20.8
19.5 to 53.5	2,748	49.0	12	0.6
	*M* = 17.8 (*SD* = 7.5)		*M* = 7.5 (*SD* = 5.3)	
	*Mdn* = 19.4 (range: 0.0–53.5)		*Mdn* = 6.1 (range: 0.0–25.0)	
Gastroenterologists per 100,000 inhabitants				
0.00 to 1.99	825	13.6	2,558	95.3
2.00 to 3.24	1,846	32.4	142	4.7
3.25 to 8.50	3,105	53.9	0	0.0
	*M* = 3.2 (*SD* = 1.2)		*M* = 0.8 (*SD* = 0.8)	
	*Mdn* = 3.3 (range: 0.0–8.5)		*Mdn* = 0.5 (range: 0.0–2.7)	
General Surgeons per 100,000 Inhabitants				
0.00 to 6.24	519	8.7	2,416	89.4
6.50 to 10.49	2,554	43.9	263	10.1
10.50 to 27.25	2,703	47.4	21	0.5
	*M* = 10.1 (*SD* = 3.1)		*M* = 4.5 (*SD* = 1.8)	
	*Mdn* = 10.4 (range: 0.0–27.2)		*Mdn* = 4.1 (range: 0.0–10.8)	
Oncologists per 100,000 inhabitants				
0.00 to 1.99	1,252	20.7	1,217	45.3
2.00 to 3.24	2,004	34.7	1,441	52.8
3.25 to 13.25	2,520	44.6	42	1.9
	*M* = 3.0 (*SD* = 1.4)		*M* = 1.6 (*SD* = 1.5)	
	*Mdn* = 3.1 (range: 0.00–9.50)		*Mdn* = 2.0 (range: 0.0–13.3)	

*Notes*. 8,476 people diagnosed with colon cancer between 1996 and 2000: 5,776 in California and 2,700 in Ontario. All between-country differences were statistically significant at *p* < .001

### Testing the Starfield-Gorey theory

Interactions are depicted in Table 2. As hypothesized, the inverse poverty-survival association was relatively strong in California (RR = 0.79, 95 % CI 0.73, 0.86), but not significant in Ontario. Consequently, among people who lived in poverty, those in Ontario were 15 percent more likely to survive for 10 years than were those in California (RR = 1.15, 95 % CI 1.02, 1.30). The Canadians were similarly advantaged when compared with their uninsured or publicly-insured counterparts in the USA (RR = 1.18, 95 % CI 1.09, 1.28). Again as hypothesized, the direct PCP-survival association was strong in Ontario (RR = 1.32, 95 % CI 1.19, 1.46), but modest in California (RR = 1.11, 95 % CI 1.00, 1.23). Consequently, in adequately supplied communities people in Ontario were 13 % more likely to survive than were their counterparts in California (RR = 1.13, 95 % CI 1.00, 1.28). High poverty neighborhoods in Ontario also had (mean = 8.6, *SD* = 2.4), on average, more than 2 more PCPs per 10,000 residents than did high poverty neighborhoods in California (mean = 6.4, *SD* = 1.8); *F* (1, 2186) = 595.77, *p* < .001.

**Table 2:** Effects of interactions of neighborhood poverty, health insurance, community physician supplies and country on 10-year survival of non-metastasized colon cancer: California and Ontario, 1996—2010

	California			Ontario				
Baseline		10-Year survival	Rate ratio		10-Year survival	Rate ratio	Canada/United States	
Observed group	Sample	Rate, %	95 % CI	Sample	Rate, %	95 % CI	Rate ratio	95 % CI
Less than 30 % vs 30 % or more of households poor in neighborhood								
Lower poverty	3,078	42.1		1,368	40.4		0.96	0.89, 1.03
High poverty	1,496	33.3	**0.79**	692	38.2	0.95	**1.15**	1.02, 1.30
			0.73, 0.86			0.86, 1.05		
Privately insured vs uninsured or publicly insured								
Private	2,065	40.8		2,060	38.7		0.95	0.88, 1.02^a^
Uninsured or public	2,509	32.8	**0.80**				**1.18**	1.09, 1.28^a^
			0.74, 0.86					
Less than 7.5 vs 7.5 or more primary care physicians per 10,000 population in community								
Lower PCP density	4,032	38.5		1,238	36.4		0.95	0.88, 1.02
High PCP density	542	42.8	**1.11**	822	48.2	**1.32**	**1.13**	1.00, 1.28
			1.00, 1.23			1.19, 1.46		
Less than 2 vs 2 or more gastroenterologists per 100,000 population in community								
Lower GE density	651	35.9		1,953	37.0		1.03	0.92, 1.16
High GE density	3,923	41.2	**1.15**	107	57.6	**1.56**	**1.40**	1.16, 1.70
			1.03, 1.28			1.28, 1.91		

*Notes.* 6,634 incident non-metastasized cases diagnosed between 1996 and 2000 were followed to 2010: 4,574 in California and 2,060 in Ontario. Rates were directly adjusted for age and stage using the California-Ontario population of cases as the standard (age categories: 25–59, 60–69, 70–79 and 80 and older; stage categories: I, II and III). Statistically significant rate ratios are bolded. The adjusted very early diagnosis (stage I) rate of 27.4 % did not differ between-countries. The adjusted 10-year survival rate of 39.2 % did not differ between-countries

^a^Both groups in the USA were compared with the Canadian group all of whom were Medicare covered

The GE supply by country interaction is depicted in the bottom of Table 2. The direct GE supply-survival association was larger in Ontario (RR = 1.56, 95 % CI 1.28, 1.91) than in California (RR = 1.15, 95 % CI 1.03, 1.28). Among those who lived in adequately supplied communities with 2 or more GEs for every 100,000 inhabitants, Ontarians were much more likely to survive than Californians (RR = 1.40, 95 % CI 1.16, 1.70). Such communities in Ontario also had (mean = 9.0, *SD* = 1.9), on average, nearly 2 more PCPs per 10,000 inhabitants than similar communities in California (mean = 7.2, *SD* = 2.0); *F* (1, 4028) = 92.40, *p* < .001.

#### Primary care precedence

Logistic regressions of predictors of 10-year colon cancer survival are displayed in Table 3. The first model shows the significant affect of PCP supply (OR = 1.60) and the interaction of PCP supply and country (OR = 1.95) on survival odds, while country itself had no affect. After these factors and noted covariates entered the model no other interactions entered. In model 2 we first entered aspects of initial colon cancer care: receipt of surgical resection, number of lymph nodes surgically harvested for pathological examination, receipt of adjuvant chemotherapy and wait times from diagnosis to surgery and from surgery to chemotherapy. The still significant affect of PCP supply (OR = 1.30) was attenuated and the PCP supply by country interaction (OR = 1.11) was no longer significant. Any Canadian primary care advantages were probably realized through the initial course of treatment. Primary care seems to have continued to offer protections in both countries throughout the years of follow-up care.

**Table 3:** Logistic regression of main effects and interaction of community primary care physician density and country on 10-year survival of non-metastasized colon cancer: California and Ontario, 1996—2010

Predictor variables	Odds ratio	95 % Confidence interval
Model 1: community primary care physician density and country		
Country	0.94	0.82, 1.09
Community primary care physician (PCP) density	**1.60**	1.30, 1.96
Community PCP density by country	**1.95**	1.50, 2.52
Model 2: colon cancer care variables entered		
Had surgical resection	**3.12**	1.64, 5.93
>15 regional lymph nodes examined	**1.23**	1.07, 1.41
Received chemotherapy	**1.28**	1.00, 1.63
Waited > 90 days for treatments^a^	0.69^b^	0.45, 1.07
Country	0.98	0.88, 1.11
Community primary care physician (PCP) density	**1.30**	1.06, 1.60
Community PCP density by country	1.11	0.82, 1.50

*Notes.* Total samples were 6,043 for model 1 and 5,908 for model 2. All effects were adjusted for age, tumor stage and grade, and place (small or large urban or rural). Gender did not enter either model so this pattern is likely the same for women and men. Statistically significant odds ratios are bolded

^a^Total wait time = wait after diagnosis for surgery + wait after surgery for chemotherapy

^b^Approached significance, 90 % confidence interval (CI) = 0.48, 1.00

## Discussion

This systematic replication of cancer care in Ontario and California found further support for the Starfield-Gorey theory of Canada-USA health care disparities. Consistent with the theory, poverty and health insurance were more predictive in the USA, community PCP supply more so in Canada. Additionally, Canada’s primary care protections were greatest among the most socioeconomically vulnerable; those who lived in poverty or were at greatest risk of being inadequately insured. In fact, PCPs were more prevalent and more effective in the poorest Canadian than in the poorest American neighborhoods. These historical observations of the protective effects of Canadian health care prior to enactment of the ACA affirmed that strengthening America’s system of primary care will probably be necessary to ensure full realization of ACA benefits. Increased privatization is an omnipresent consideration in Canada. This study also strongly suggested that adding a second, private tier to Canadian health care would only serve to reproduce the socioeconomic disparities observed in the USA. Canadian patients covered by single payer Medicare did as well as privately insured patients in the USA.

Gastroenterologist supply was also relatively more protective in Ontario than California. In absolute terms, however, its impact was probably much greater in California where most study participants lived in adequately supplied communities. Few Ontario participants lived in such communities. Additionally, PCPs were more prevalent in high gastroenterologist density Canadian communities than they were in similarly well-supplied American communities. The case mix of adequate physician supplies, primary care and key specialists, seemed potentiating. Protections attributable to PCP supply probably reinforce the protective effects of gastroenterologist supply. However, primary care took precedence as it explained most of the Canadian survival advantage we observed. This study systematically replicated evidence of a more effective primary care system in Canada. Its policy implications are: (1) chances for the success of health insurance reforms in the USA will be greatly increased by concomitant reforms to strengthen primary care and (2) Canada’s primary care system ought to be maintained and strengthened in the few places where it may be vulnerable [53–55].

Our research is consistent with that suggesting Medicaid expansion may not be enough to ensure access among the poor or near poor [Gorey KM et al., unpublished observations, 2015, 56]. Oregon’s Medicaid experiment found that expansion caused an increase in emergency room visits, including visits for conditions treatable by PCPs. It also increased PCP office visits as well as the use of preventive services and it drastically reduced catastrophic health care expenditures [57, 58]. An adequate supply of participating PCPs seems central. Prospective study of primary care in Canada along with primary care trends in diverse post-ACA communities across America will substantially augment trial findings and identify ways to maximize ACA protections. Prospective study of specialized care, gastroenterology for colon cancer care [59–62] and diverse specialist physicians for other health domains will also be needed to fully inform physician supply policies in both countries.

### Future research needs

The primary care-based Canadian advantage on survival was largely mediated by access to a more effective initial course of treatment, typically provided during the first six months to a year after diagnosis. But primary care seemed to continue to be beneficial in both countries throughout this study’s remaining nine years of follow-up. This is consistent with studies in Canada and the USA that have observed associations between PCP visits and high quality follow-up care of cancer survivors [63–66]. The cancer registries we studied, however, did not provide data on follow-up care so we were not able to study the effects of poverty, insurance and physician supplies on longer term, follow-up care. Such studies are needed to advance understandings of the entire colon cancer care process and to facilitate evidence-based decision making and policy planning in the post-ACA era in both countries.

Our research group has systematically replicated and affirmed the Starfield-Gorey theory for Canada-USA comparisons of colon and breast cancer care. We invite independent research teams to test the theory across other health care processes and outcomes. Such knowledge will help policy makers to empirically plan future PCP and specialist physician supplies, enabling the highest quality health care. We continue to study cancer care among those who live in poverty, now prospectively in diverse, including so-called “red” and “blue” states.

### Potential limitations

Our findings may not be generalizable to all Americans and Canadians. However, because one in three Canadians lives in Ontario and one in ten Americans lives in California, we think they have ample external validity [67, 68]. Admittedly, we purposefully oversampled those who lived in poverty so our findings are most representative of their experiences. Furthermore, California’s expanded Medicaid program is more liberal than most states’ so estimates of health care inequities among the poor there are probably underestimates of the nation’s [69–71].

Some may question our choice of overall, rather than cancer-specific survival. Our rationales follow. First, cancer seemed the underlying cause of death among the vast majority of participants in Ontario and California. However, although vital status and length of survival are highly accurate in North American cancer registries, the underlying cause of death is not [37, 72–74]. We avoided bias that would intrude through such, often substantial, death certificate error by focusing on overall survival. Second, the underlying cause of deaths not identified as cancer deaths can still be associated with lack of treatment or with their complications [75]. Furthermore, overall survival is the gold standard in most trials and the principle that guides most oncologic practice [76, 77]. Recall that participants were diagnosed with non-metastasized disease, imminently treatable in most cases. It seems likely that the guiding principle among most participants and their physicians was to maximally extend life. Therefore, we think overall survival is the most policy-telling, practical indicator of clinical and human significance. Finally, because our analysis had a hierarchical structure, with individuals nested within neighborhoods and counties, some readers may wonder why we did not use a multi-level regression model. Each key neighborhood and county-based variable was constituted by only two clusters. And germinal literature concurs that multi-level models should not be estimated with data consisting of fewer than 10 clusters. There has been some debate about the exact criterion of “too few,” but sensitivity analyses strongly suggested to us that two would definitely be too few [78, 79]. Other potential limitations have been discussed [13, 16, 52].

## Conclusions

As the Starfield-Gorey theory hypothesized, poverty and health insurance were more predictive in the USA, community physician supplies more so in Canada. Canada’s primary care protections were greatest among the most socioeconomically vulnerable. This historical study of the protective effects of Canadian health care prior to enactment of the ACA clearly suggested that notwithstanding the importance of insuring all, strengthening America’s system of primary care will probably be the best way to ensure that the ACA’s full benefits are realized. As for Canada, its strong primary care system ought to be maintained.

### Human participants ethical review

This study was reviewed and cleared by the University of Windsor’s research ethics board.

## Abbreviations

ACA: Affordable care act
CCO: Cancer Care Ontario
CDPH: California Department of Public Health
CI: Confidence interval
CIHI: Canadian Institute for Health Information
CRGC: Cancer Registry of Greater California
GE: Gastroenterologist
NCI: National Cancer Institute
OR: Odds ratio
PCP: Primary care physician
RLN: Regional lymph node
RR: Rate ratio
USA: United States of America
